# Tumor differentiation-dependent conditional survival of patients with operable thyroid cancer

**DOI:** 10.3389/fendo.2024.1446312

**Published:** 2024-11-14

**Authors:** Ruo-nan Yu, Zi-qi Zhang, Ping Zhang, Hao Zhang, Hui-ling Qu, Wen-wu Dong

**Affiliations:** ^1^ Department of Thyroid Surgery, The First Hospital of China Medical University, Shenyang, China; ^2^ Clinical Medicine, Innovation Institute, China Medical University, Shenyang, China; ^3^ Department of Neurology, The General Hospital of Northern Theater Command, Shenyang, China

**Keywords:** thyroid cancer, surgery, tumor differentiation, prognosis, hazard, conditional survival

## Abstract

**Objective:**

Little is known about the changing risk profile of death and conditional survival in patients with operable thyroid cancer. This study aimed to investigate the annual hazard rate of cancer death, actuarial disease-specific survival (DSS), and conditional DSS in patients with thyroid cancer and explore the effects of tumor differentiation.

**Methods:**

Patients diagnosed with thyroid cancer (N = 132,354) between 2004 and 2019 were identified from the Surveillance, Epidemiology, and End Results database. The hazard function was used to estimate the annual hazard rate of death. The Kaplan-Meier method and log-rank test were used for the calculation and between-group comparison of actuarial DSS, respectively. The life table was used to estimate the conditional DSS.

**Results:**

A total of 1896 (1.4%) patients died due to thyroid cancer during the follow-up period. Patients with ATC (68.9%, 313/454) were more likely to die than those with PDTC (19.4%, 171/883) or DTC (1.1%, 1412/131017). For the entire cohort, patients with DTC and PDTC had excellent and relatively stable one-year conditional survival, respectively; patients with ATC had the worst one-year conditional survival, but they achieved the greatest improvements. The worst one-year conditional survival and the most obvious improvement were seen in patients with ATC regardless of any SEER Summary Stage.

**Conclusion:**

Prognosis improved over time in a tumor differentiation-dependent manner in patients with operable thyroid cancer after diagnosis. This information provides more precise dynamic evaluations of the long-term prognosis of thyroid cancer survivors and paramount clinical implications for individualized treatment and surveillance.

## Introduction

Thyroid cancer is the most common endocrine malignancy (1) and has significantly increased worldwide over the past few decades (2, 3). Based on the degree of tumor differentiation, thyroid cancers are categorized as differentiated thyroid cancer (DTC), poorly-differentiated thyroid cancer (PDTC)/differentiated high-grade thyroid cancer (DHGTC) and anaplastic thyroid cancer (ATC). DHGTC is considered as a distinct pathological entity in the latest World Health Organization (WHO) histologic classification of thyroid neoplasms released in 2022 (4). DTC, which encompasses papillary cancer, follicular cancer and oncocytic cancer (previously known as Hürthle cell cancer), accounts for approximately 90% of all diagnosed thyroid cancers and generally has a slow progression and good prognosis with a 10-year survival rate of more than 90% (5, 6). PDTC and ATC, although rare, are the most clinically aggressive thyroid cancers. Patients with PDTC has a 10-year survival rates of 34-50%, while very few patients with ATC can survive longer than 1 year (7, 8). The clinical outcome of DHGTC is similar to PDTC, showing an intermediate prognosis between DTC without high-grade features and ATC (9).

Curative surgery is the cornerstone of the treatment for patients with thyroid cancer. Patients with thyroid cancer of different tumor differentiation might want to be informed about a realistic view of life expectancy after surgery. The survival rates of patients with thyroid cancer after surgery are known, but the death hazard is not constant and the likelihood of survival could change over time during follow-up. Thus, the time-dependent risk of death and survival probability for a patient who has already survived a long time after surgery remains to be further studied.

Traditional survival estimates mainly focus on survival rates at a given time and cannot provide accurate and dynamic prognostic evaluations over time. Time-dependent survival analysis, such as annual death hazard and conditional survival, can reflect real-time changes in death risk or survival at a specific time point and provide a more accurate and dynamic outlook of prognosis. The annual death hazard illustrates the absolute hazard of death at any instant among the remaining at-risk individuals (10). The conditional survival represents the probability of surviving certain additional years for patients who have lived for a designated period (11). These dynamic prognostic data provide important clinical implications for personalized treatment and surveillance. Furthermore, little evidence known about the prognostic factor of subtype and histological grade, since the prognostic impact has always been distinguished between DTC vs. ATC. The AJCC/UICC TNM, QTNM, AGES, MACIS and AMES systems consider the histological type (differentiated vs anaplastic), AGES also considers the tumor grade. A nomogram with excellent performance was developed for predicting the probability of death for patients with thyroid cancer based on a competing risks model and histological subtype was also considered as an important clinical predictive factor (13). All of these indicate the importance of histological subtype and grade in the prognosis of thyroid cancer. Thus, this study aimed to investigate the annual hazard rate of cancer death, actuarial disease-specific survival (DSS), and conditional DSS in thyroid cancer patients, and further explore the effects of tumor differentiation using the Surveillance, Epidemiology, and End Results (SEER) database.

## Materials and methods

Patient were selected between 2004 and 2019 from the SEER database, which was maintained by the National Cancer Institute of the USA. It currently covers and publishes cancer incidence and survival data of approximately 34.6% of the US population across 18 regions. Patients diagnosed with DTC, PDTC or ATC were identified as per the International Classification of Diseases for Oncology, 3rd Edition (ICD-O-3) histological codes. The exclusion criteria were as follows: i) not undergoing thyroidectomy, ii) more than one type of primary malignancy, iii) unknown SEER combined summary stage, iv) patients with DHGTC and v) zero days of follow-up. The following information was extracted: age at diagnosis, sex, race/ethnicity, and the combined summary stage (2004+). This database contains anonymized patient information and is publicly accessible. Informed consent and approval from the institutional review boards were not required. The work has been reported in line with the Strengthening the Reporting of Cohort Studies in Surgery (STROCSS) reporting guidelines (12).

### Statistical analysis

The DSS was measured from the time of diagnosis to death due to PTC as the primary endpoint of this study. The maximum likelihood estimate from the piecewise exponential model was used to evaluate the annual hazard rate, and the kernel-smoothing method was used to display the graphics. The Kaplan-Meier method and log-rank test were used for the calculation and between-group comparison of actuarial DSS, respectively. The conditional survival was defined as the probability of surviving additional y years, given that a patient has already lived for x years, and can be stated as follows: CS(x|y) = S(x+y)/S(x), where S(x) represents overall survival at x years. The conditional survival was computed from the life table survival data. A 2-tailed P value < 0.05 was considered statistically significant. All analyses were conducted using Stata software (version 16.0; Stata Corporation Ltd., College Station, TX, USA).

## Results

### Patient characteristics

Baseline characteristics of the study cohort are summarized in **Table 1**. A total of 132,354 patients undergoing surgery for thyroid cancer were enrolled for analysis. Overall, 131,017 (99.0%) patients had DTC. Only 883 (0.7%) and 454 (0.3%) patients had PDTC and ATC, respectively. The median age at diagnosis was 47 years (interquartile range, 36-58 years). The lower the degree of tumor differentiation, the older the patients (47 years for DTC, 55 years for PDTC and 67 years for ATC). Females and white predominated regardless of type of thyroid cancer. Localized disease (68.8%) was common in DTC, while distant metastasis (58.6%) was common in ATC. PDTC lied between DTC and ATC. The median follow-up duration was 74 months (interquartile range, 35-121 months).

**Table 1:** Baseline characteristics of patients with operable thyroid cancers.

Characteristics	DTC n= 131,017, n (%)	PDTC n=883, n (%)	ATC n=454, n (%)	Total n=132,354, n (%)
Age (median, years)	47 (36-58)	55 (42-66)	67 (59-75)	47 (36-58)
Sex				
Female	101,918 (77.8)	560 (63.4)	268 (59.0)	102,746 (77.6)
Male	29,099 (22.2)	323 (36.6)	186 (41.0)	29,608 (22.4)
Race/ethnicity				
White	104,838 (80.0)	672 (76.1)	369 (81.3)	105,879 (80.0)
Black	8,259 (6.3)	84 (9.5)	35 (7.7)	8,378 (6.3)
Others/unknown	17,920 (13.7)	127 (14.4)	50 (11.0)	18,097 (13.7)
Tumor stage				
Localized	90,090 (68.8)	383 (43.4)	48 (10.6)	90,521 (68.4)
Regional	37,931 (29.0)	334 (37.8)	140 (30.8)	38,405 (29.0)
Distant metastasis	2,996 (2.3)	166 (14.5)	266 (58.6)	3,428 (2.6)

DTC, differentiated thyroid cancer; PDTC, poorly differentiated thyroid cancer; ATC, anaplastic thyroid cancer; Tumor stage was categorized according to SEER Combined Summary Stage (2004+).

### Death hazard analysis

**Figure 1:**
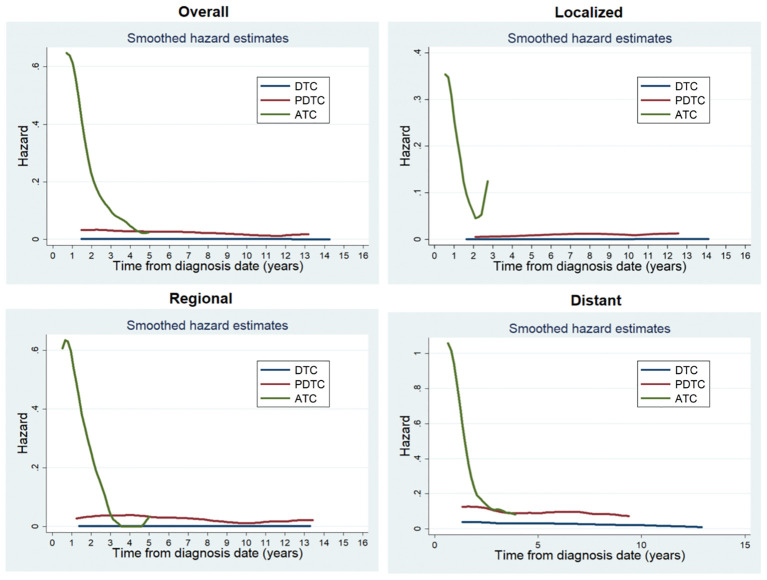
Annual hazard rates of cancer death of patients with different tumor differentiation for the entire cohort and at different SEER Summary Stages.

A total of 1896 (1.4%) patients died due to thyroid cancer during the follow-up period. Patients with ATC (68.9%, 313/454) were more likely to die than those with PDTC (19.4%, 171/883) or DTC (1.1%, 1412/131017). For the entire study population, the annual hazard curve of cancer mortality of patients with ATC showed a downward trend from fast to slow without an obvious peak, while that of patients with PDTC or DTC showed two almost parallel lines. The death hazard rate of patients with ATC was obviously higher than that of patients with PDTC or DTC. The pattern of death hazard of patients with different tumor differentiation was almost similar to that of the entire study population regardless of any SEER Summary Stage (**Figure 1**).

### Traditional actuarial DSS

**Figure 2:**
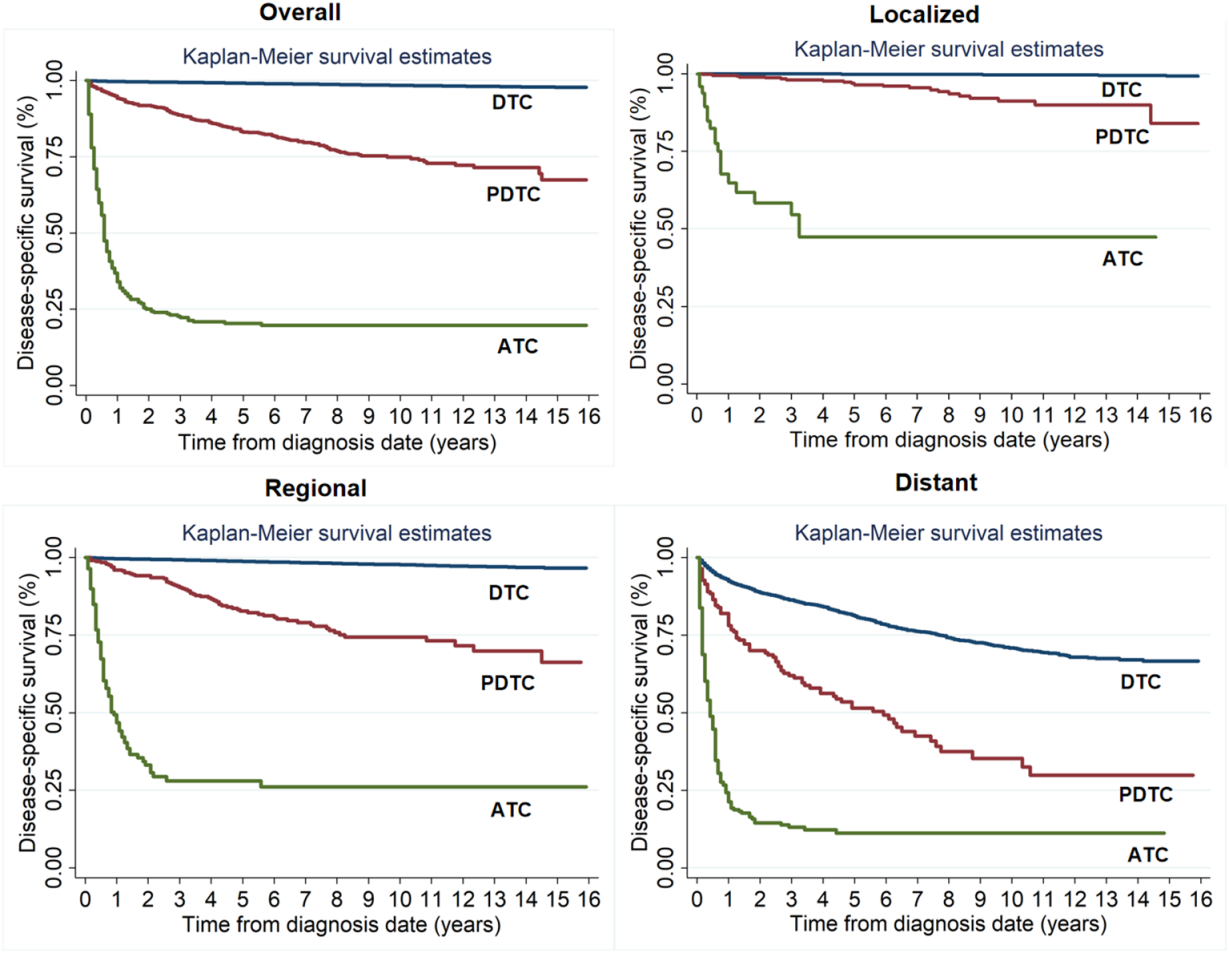
Kaplan-Meier survival estimates for actuarial disease-specific survival of patients with different tumor differentiation for the entire cohort and at different SEER Summary Stages.

The 1- and 10-year DSS rates were 36.81% ± 2.42% and 19.82% ± 2.33% for ATC, 94.95% ± 0.78% and 74.92% ± 1.96% for PDTC, and 99.76% ± 0.20% and 98.47% ± 0.25% for DTC, respectively. The prognosis of patients with ATC was worst, followed by PDTC and DTC. Moreover, regardless of any SEER Summary Stage, patients with ATC had worse DSS compared to those with PDTC or DTC. The higher the SEER Summary Stage, the closer the prognosis of PDTC was to ATC (**Figure 2**).

### Conditional DSS

**Figure 3:**
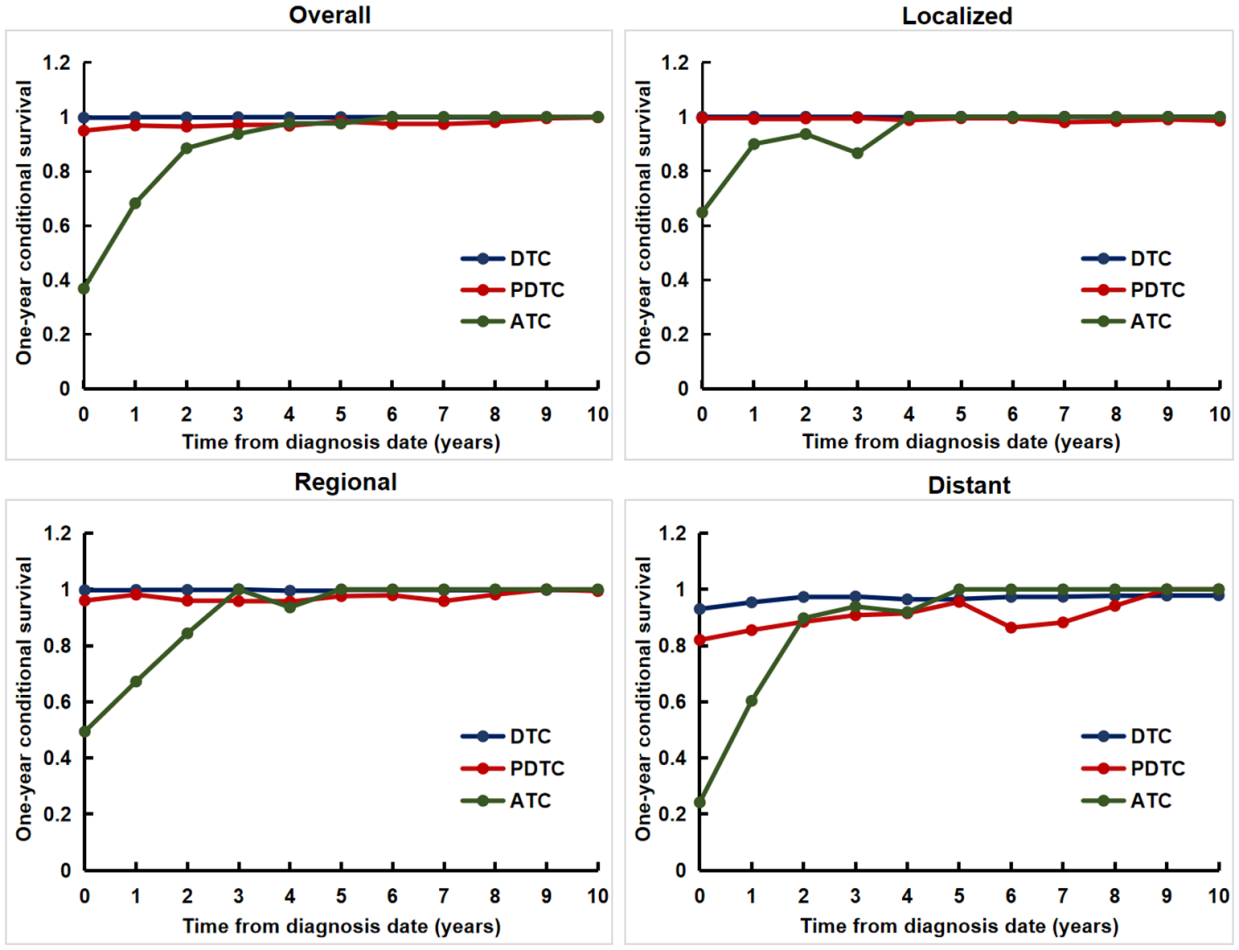
The one-year conditional disease-specific survival for patients with different tumor differentiation for the entire cohort and at different SEER Summary Stages.

For the entire cohort, patients with DTC and PDTC had excellent and relatively stable one-year conditional survival, respectively; patients with ATC had the worst one-year conditional survival, but they achieved the greatest improvements. For patients with different SEER Summary Stages, patients with distant disease had the worst one-year conditional survival, but obtained the most significant improvement. The worst one-year conditional survival and the most obvious improvement were seen in patients with ATC regardless of any SEER Summary Stage (**Figure 3**).

## Discussion

In this study, we identified tumor differentiation has a significant impact on the prognosis of patients with operable thyroid cancer. For the entire study population, the death hazard rate of patients with ATC was obviously higher than that of patients with PDTC or DTC, showing a downward trend from fast to slow without an obvious peak. The prognosis of patients with ATC was worst, followed by PDTC and DTC. Patients with DTC and PDTC had excellent and relatively stable one-year conditional survival, respectively; patients with ATC had the worst one-year conditional survival, but they achieved the greatest improvements. Moreover, we achieved the same results when conducting stratified analysis based on the SEER Summary Stage. In brief, we demonstrated that improved survival probability was strongly dependent on tumor of the prognosis irrespective of where they are in any SEER Summary Stage. Our study is the first large population-based study comparing dynamic outcomes in thyroid cancer patients with different tumor differentiation treated with surgery.

Surgical resection remains the cornerstone of treatment for patients with thyroid cancer and this explains the improved outcome of patients who received surgery in our study. Previous studies have reported on the survival outcomes of patients with different tumor differentiation (6, 14). The prognosis of patients with DTC is usually good with the postoperative 10-year survival rate exceeding 90% (6, 14). However, PDTC and ATC have much worse outcomes. Lee et al. analyze the temporal changes of the long-term outcomes of 184 patients who were diagnosed with ATC or PDTC with 38.9 months. They found that the 5-year DSS rate was significantly higher in PTDC than ATC among all patients (65.8% vs.14.3%) and also in patients with resectable tumors (71.4% vs. 26.5%) (15). A recent epidemiological study from Denmark using a national cancer registry also reported that the 5-year survival rates were 91.1% and 79.9% in PTC and FTC, respectively, 63.6% in PDTC and 12.2% in ATC (16). We have achieved similar results. Not only that, our study clearly demonstrated dynamic prognostic changes of thyroid cancer patients with different tumor differentiation.

In previous studies dynamic evaluation of prognosis for malignant tumors have mainly been conducted based on SEER stage, TNM, or clinical pathological characteristics (17–20). Wang et al. found that gastric cancer patients with a higher stage from the SEER database had lower 5-year conditional survival, but the greatest increase in conditional survival occurred as more time elapsed from diagnosis (17). Shin et al. estimated the 5-year conditional survival of patients with ovarian cancer between 1997 and 2016 using data from the Korean Central Cancer Registry and found that the 5-year conditional survival improved over time, and the largest improvements were noted in patients with poorer initial prognostic factors (e.g., higher cancer stage) (18). Wang et al. investigate the CS and dynamic failure hazard in non-metastatic nasopharyngeal cancer receiving intensity-modulated radiotherapy and found that survival prognosis of non-metastatic non-metastatic nasopharyngeal cancer evolves over time with distinct dynamic patterns across TNM stages (19). Ploussard et al. evaluated the changes in the 5-year conditional survival rates of 8,141 patients treated with radical cystectomy for bladder cancer at 15 international academic centers between 1979 and 2012, and found that the 5-year conditional survival improved mainly for surviving patients with advanced-stage disease (20). These results suggest that the prognosis improved over time, and the largest improvements were noted in patients with higher stage or poorer initial prognostic factors. Our results indicated that the prognosis improved over time in a tumor differentiation-dependent manner among patients with thyroid cancer. The most obvious improvements were found in patients with ATC.

The strengths of our study include its large cohort with a sufficient sample size, nationally representative population, and long-term follow-up. However, this study had several limitations. First, treatment information was limited, and it is possible that changes in treatment regimens during the study period. Second, the study is subject to U.S. population, and the findings may not be generalizable to other populations. Third, data on tumor recurrence were not available, and we were unable to assess recurrence-free survival.

## Conclusion

This nationwide study described the hazard rate of cancer death, traditional actuarial DSS, and conditional DSS of patients with operable thyroid cancer of different tumor differentiation, providing a dynamic prognostic evaluation for these patients. Understanding the hazard rate and the conditional survival of thyroid cancer is key to creating more tailored treatment plans and surveillance.

## Data Availability

The datasets presented in this study can be found in online repositories. The names of the repository/repositories and accession number(s) can be found below: All data used in this study can be freely accessed from the SEER program (https://seer.cancer.gov/).
